# Genomic Epidemiology, Antimicrobial Resistance, and Virulence of Clinical *Enterococcus* Isolates from Hat Yai, Thailand: Genomic Relatedness and Lineage Persistence

**DOI:** 10.3390/antibiotics15090867

**Published:** 2026-09-06

**Authors:** Wajeeorn Ouancharee, Sareenee Saleah, Rattanaruji Pomwised, Arnon Chukamnerd, Komwit Surachat, Rosesathorn Soontarach, Kamonnut Singkhamanan, Sarunyou Chusri

**Affiliations:** 1 Division of Infectious Disease, Department of Internal Medicine, Faculty of Medicine, Prince of Songkla University, Songkhla 90110, Thailand; wajeeorn.och@gmail.com; 2 Department of Biomedical Sciences and Biomedical Engineering, Faculty of Medicine, Prince of Songkla University, Songkhla 90110, Thailand; sareeneesaleah@gmail.com (S.S.); komwit.s@psu.ac.th (K.S.); skamonnu@medicine.psu.ac.th (K.S.); 3 Division of Biological Science, Faculty of Science, Prince of Songkla University, Songkhla 90110, Thailand; rattanaruji.p@psu.ac.th; 4 Department of Microbiology, Faculty of Science, King Mongkut’s University of Technology Thonburi, Bangkok 10140, Thailand; arnonchukamnerd@hotmail.com; 5 Department of Science, Technology and Innovation, Faculty of Science, Chulabhorn Royal Academy, Bangkok 10210, Thailand; rosesathron_pim@hotmail.com

**Keywords:** *Enterococcus faecalis*, *Enterococcus faecium*, vancomycin-resistant enterococci, daptomycin resistance, multidrug resistance, virulence factor, biofilm formation, whole-genome sequencing, spatiotemporal analysis

## Abstract

**Background/Objectives**: *Enterococcus* species are important causes of healthcare-associated infections, particularly multidrug-resistant (MDR) and vancomycin-resistant enterococci (VRE). This study characterized antimicrobial susceptibility, biofilm formation, genomic features, and molecular epidemiology of clinical *Enterococcus* isolates from southern Thailand. **Methods**: Forty clinical isolates, comprising *E. faecalis* (*n* = 22) and *E. faecium* (*n* = 18), were characterized by antimicrobial susceptibility testing, biofilm assays, and whole-genome sequencing. **Results**: All isolates were susceptible to linezolid and oritavancin, whereas non-susceptibility to ampicillin, fosfomycin, vancomycin, and daptomycin was 60.0%, 57.5%, 12.5%, and 5.0%, respectively. High-level gentamicin resistance and MDR occurred in 37.5% and 22.5%, respectively. Strong biofilm formation predominated in *E. faecalis* (86.4%), whereas 94.4% of *E. faecium* were weak producers. ST6 and ST17 predominated in *E. faecalis* and *E. faecium*, respectively. All five VRE isolates were *E. faecium* ST17 or ST262 and carried *vanA*. High-level ampicillin-resistant *E. faecium* harbored the PBP5 S466′ insertion and multiple C-terminal substitutions. To our knowledge, this is the first report in Thailand of both daptomycin-resistant *E. faecium* and resistance-associated LiaS T120A and LiaR W73C substitutions. Closely related isolates within *E. faecalis* ST6 and *E. faecium* VRE ST17, ST117, and ST262 showed spatiotemporal patterns consistent with potential epidemiological linkage or lineage persistence. Three novel STs were identified, with *E. faecium* ST3096 exhibiting high-level ampicillin resistance, fosfomycin resistance, and reduced daptomycin susceptibility. **Conclusions**: These findings reveal emerging resistant lineages and spatiotemporal patterns of genomic relatedness, supporting continued genomic surveillance of clinical enterococci in Thailand.

## 1. Introduction

*Enterococcus* spp. are Gram-positive, facultatively anaerobic commensals of the human gastrointestinal tract that have emerged as major opportunistic pathogens in healthcare settings. These organisms are important causes of urinary tract infections (UTIs), bloodstream infections, infective endocarditis, intra-abdominal infections, surgical site infections, and catheter-associated infections [1,2]. Among the recognized *Enterococcus* species, *Enterococcus faecalis* and *Enterococcus faecium* cause most human infections and together account for approximately 90% of enterococcal bloodstream infections [3]. Their ability to tolerate environmental stress, persist on hospital surfaces, form biofilms, and acquire antimicrobial resistance (AMR) determinants contributes substantially to their success as healthcare-associated pathogens [1,4].

AMR in enterococci poses a major clinical challenge by limiting effective therapeutic options. Hospital-adapted *E. faecium* lineages are especially problematic due to their frequent resistance to ampicillin (AMP), vancomycin (VAN), and high-level aminoglycosides, substantially limiting therapeutic options [2,5,6]. VAN-resistant *E. faecium* (VREfm) continues to represent a major public health concern and is classified as a high-priority pathogen in the 2024 WHO Bacterial Priority Pathogens List [7]. Recent global surveillance reported substantial geographical variation in VAN resistance among *E. faecium*, with the highest regional rate observed in North America (53.3%) [8]. Linezolid (LZD) and daptomycin (DAP) consequently remain important antimicrobial agents for severe VRE infections, although resistance to these agents has been reported [9]. Although LZD resistance remains relatively uncommon, reaching a maximum of 2.9% in Asia, DAP resistance among *E. faecium* isolates shows greater regional variation, ranging from 3.3% to 13.4% [8]. Collectively, these resistance patterns highlight the complexity of enterococcal AMR and the need to understand its underlying molecular mechanisms.

Multiple genetic mechanisms contribute to AMR in enterococci. VAN resistance is primarily mediated by acquired *van* gene clusters, particularly the *vanA* and *vanB* types, whereas high-level aminoglycoside resistance is commonly mediated by aminoglycoside-modifying enzymes [1,5]. In *E. faecium*, high-level AMP resistance is strongly associated with sequence variation and structural alterations in the low-affinity penicillin-binding protein 5 (PBP5), particularly within its C-terminal transpeptidase domain [10]. Reduced susceptibility or resistance to DAP involves alterations in the cell-envelope stress response and membrane phospholipid homeostasis, particularly involving the LiaFSR regulatory system and cardiolipin synthase [11,12]. LZD resistance is associated with mutations in the 23S rRNA and the acquisition of transferable resistance determinants, including *cfr*, *optrA*, and *poxtA* [5,12].

In addition to AMR, biofilm formation and virulence-associated determinants contribute to the persistence and pathogenicity of enterococci. Biofilms enhance adherence to host tissues and medical devices and can protect bacterial populations from antimicrobial exposure, environmental stresses, and host immune responses [13]. Clinical *E. faecalis* isolates have been reported to exhibit greater biofilm-forming ability than *E. faecium* [14]. Several virulence factor genes (VFGs), including *esp*, *ace*, *asa1/prgB*, *ebpABC*, *gelE*, and *fsrABC*, have been implicated in adhesion and biofilm development [13]. However, biofilm formation is multifactorial and may not be predicted by individual VFGs alone [13,14], highlighting the importance of evaluating virulence genotypes alongside phenotypic biofilm-forming ability.

Genomic epidemiological studies are increasingly valuable for understanding the circulation and persistence of *Enterococcus* lineages in healthcare settings. Whole-genome sequencing (WGS), particularly when integrated with patient-level spatiotemporal data, enables high-resolution assessment of genomic relatedness and potential epidemiological linkages [15,16]. In Thailand, molecular epidemiological studies have documented the circulation of VREfm in hospitals. A previous study from southern Thailand demonstrated the persistence and genetic relatedness of VREfm isolates in a tertiary-care hospital using molecular typing [17]. More recent WGS-based studies have revealed the genomic diversity and distribution of high-risk VREfm lineages, including sequence type 17 (ST17) and other hospital-associated STs, across different regions of Thailand [18,19,20].

However, high-resolution genomic epidemiological studies integrating genomic relatedness with patient-level spatiotemporal data remain limited, particularly for clinical *E. faecalis* and *E. faecium* in southern Thailand. Therefore, this study characterized clinical isolates of both species using integrated phenotypic and genomic approaches. WGS was used to determine STs, AMR and VF determinants, resistance-associated mutations, and core-genome single-nucleotide polymorphism (cgSNP) relationships, with clinical and spatiotemporal data incorporated to evaluate potential epidemiological linkages and lineage persistence.

## 2. Results

### 2.1. Clinical Sources and Patient Demographics of Enterococcus spp. Isolates

A total of 40 clinical *Enterococcus* isolates were included in the study, comprising 22 *E. faecalis* (55%) and 18 *E. faecium* (45%) isolates. The clinical metadata showed that all *E. faecalis* isolates originated from Hat Yai Hospital, whereas 16 *E. faecium* isolates were from Hat Yai Hospital and two were from Songklanagarind Hospital. The length of hospital stay ranged from 2 to 69 days for *E. faecalis* (median, 16 days) and from 4 to 43 days for *E. faecium* (median, 18 days). Almost all isolates (39/40, 97.5%) were collected >48 h after admission (Table S1).

Most isolates were collected from urine specimens (72.5%, 29/40), followed by blood and pus (12.5%, 5/40) (Table 1). Urine was the predominant source for both species with more common among *E. faecalis* isolates (86.4%, 19/22) than *E. faecium* isolates (55.6%, 10/18). Notably, most blood isolates (80%, 4/5) were *E. faecium*, whereas only one was *E. faecalis*.

Regarding host demographics, most *Enterococcus* isolates were from female patients 57.5% (23/40). Among *E. faecalis* isolates, females represented 63.6% (14/22) of cases and males 36.4% (8/22). Whereas among *E. faecium* isolates, both females and males accounted for 50.0% (9/18) of cases. Patient ages ranged from < 1 to 94 years, with median ages of 59 and 69 years for *E. faecalis* and *E. faecium* infections, respectively. Overall, the clinical data indicated that enterococcal infections were predominantly associated with urinary tract specimens and occurred mainly among older adults.

### 2.2. Antimicrobial Susceptibility Profiles

All *Enterococcus* spp. isolates were susceptible to LZD and oritavancin (ORI). Non-susceptibility to AMP, fosfomycin (FOS), and VAN was observed in 60.0% (24/40), 57.5% (23/40), and 12.5% (5/40) of isolates, respectively (Table 2). High-level gentamicin (GEN) resistance (HLGR) was detected in 37.5% (15/40) of the isolates. For DAP, almost all *E. faecalis* isolates were susceptible, except for one (4.5%, 1/22) that was intermediate. Among *E. faecium*, 17 isolates (94.4%) were classified as susceptible-dose dependent (SDD), including 13 with minimum inhibitory concentrations (MICs) < 4 µg/mL and four with MICs of 4 µg/mL (Table S1). Only one *E. faecium* isolate was DAP-resistant.

Interestingly, all *E. faecium* isolates exhibited high-level AMP resistance (100%, 18/18), with MICs > 128 µg/mL. In contrast, AMP susceptibility predominated among *E. faecalis* isolates (72.7%, 16/22), while AMP-resistant *E. faecalis* isolates had MICs ranging from 16 to 32 µg/mL. FOS non-susceptibility and HLGR were more prevalent in *E. faecalis* than in *E. faecium* whereas VRE were detected exclusively in *E. faecium*.

MIC distributions were consistent with the observed antimicrobial susceptibility patterns (Table 3). *E. faecium* showed substantially higher AMP MICs than *E. faecalis*, with MIC_50_/MIC_90_ values of >128/>128 and 4/32 µg/mL, respectively. Notably, VAN MICs were substantially higher in *E. faecium* than in *E. faecalis*, with MIC_50_/MIC_90_ values of 1/128 and 1/2 µg/mL, respectively. DAP MIC_50_/MIC_90_ values were also higher in *E. faecium* (2/4 µg/mL) than in *E. faecalis* (0.5/2 µg/mL). High GEN MICs were observed in both species, particularly in *E. faecalis* (MIC_50_, 1024 µg/mL) and *E. faecium* (MIC_90_, >1024 µg/mL). For FOS, MIC_50_ values were 128 and 64 µg/mL in *E. faecalis* and *E. faecium*, respectively, while the MIC_90_ was 512 µg/mL for both species. In contrast, LZD and ORI demonstrated relatively low MICs in both species.

### 2.3. Distribution of Multidrug Resistance (MDR) Patterns

The AMR patterns of the 40 *Enterococcus* isolates are shown in Table 4. Among *E. faecalis* (*n* = 22), the most common AMR pattern was FOS (31.8%, 7/22), followed by AMP–HLGR–FOS (22.7%, 5/22). Among *E. faecium* (*n* = 18), the most frequent AMR pattern was AMP (33.3%, 6/18), followed by AMP–FOS (22.2%, 4/18).

Overall, three MDR patterns were identified among 22.5% (9/40) of the isolates. The predominant resistance pattern was AMP–HLGR–FOS (15.0%, 6/40), with five of six isolates being *E. faecalis*. The AMP–VAN–HLGR pattern was the second most common, detected in two *E. faecium* isolates (5.0%, 2/40). The AMP–DAP–FOS pattern was detected in only one *E. faecium* isolate. Notably, two MDR isolates were VRE.

### 2.4. Biofilm-Forming Ability

The distribution of biofilm-forming categories among the 40 clinical *Enterococcus* isolates is summarized in Table 5. Of these, 20 isolates (50.0%) were strong biofilm producers, three (7.5%) as moderate producers, and 17 (42.5%) as weak producers. No non-biofilm producers were identified.

Biofilm-forming ability was significantly higher in *E. faecalis* than in *E. faecium* (median OD_570_, 1.017 vs. 0.160; *p* < 0.001). Among the *E. faecalis* isolates, 19 (86.4%) were strong biofilm producers and three (13.6%) were moderate producers. In contrast, among the *E. faecium* isolates, 17 (94.4%) were weak biofilm producers, while only one (5.6%) was a strong producer. No moderate or non-biofilm-producing *E. faecium* isolates were identified.

### 2.5. Genome Characteristics and Pan-Genome Profiles

The *E. faecalis* genomes ranged from approximately 2.66 to 3.44 Mb and contained 2508–3607 coding sequences (CDSs), with GC contents ranging from 36.98% to 37.71%. In comparison, *E. faecium* genomes were more uniform in size, ranging from approximately 2.73 to 3.03 Mb, with 2764–3121 CDSs and GC contents of 37.54–37.92%.

Despite relatively consistent GC content within each species, *E. faecalis* showed greater variation in genome size but generally more contiguous assemblies than *E. faecium* (Table S2). The *E. faecalis* assemblies comprised 15–146 contigs with L50 values of 1–8, whereas *E. faecium* assemblies were more fragmented, comprising 164–274 contigs with L50 values of 18–29.

Pan-genome analysis of 22 *E. faecalis* and 18 *E. faecium* isolates revealed distinct gene-content profiles (Figure S1). The *E. faecalis* pan-genome comprised 5618 gene clusters, including 2125 core genes (37.8%) and 3493 accessory genes (62.2%). In comparison, *E. faecium* comprised 4408 gene clusters, including 1909 core genes (43.3%) and 2499 accessory genes (56.7%). The predominance of accessory genes in both species indicates considerable gene-content diversity among the studied isolates.

### 2.6. Distribution of Molecular Types

A total of 14 STs were identified among *E. faecalis* isolates. ST6 was the predominant type (9/22, 40.9%), whereas each of the remaining 13 STs was represented by a single isolate. These included ST16, ST21, ST41, ST64, ST67, ST81, ST116, ST179, ST387, ST506, ST1886, and two novel STs (ST2181 and ST2183). ST2183 (isolate E11) was identified as a single-locus variant of ST6 and harbored a novel allele in the *gki* gene. In contrast, ST2181 (isolate E38) exhibited a distinct allelic profile and was not closely related to other identified STs. In addition, three capsular polysaccharide (CPS) genotypes were identified, including CPS1 (31.8%), CPS2 (54.5%), and CPS5 (13.6%). CPS2 was the most prevalent genotype consisted of ST6, ST16, ST116, and ST2183. In contrast, CPS1 exhibited greater ST diversity, comprising ST21, ST41, ST64, ST67, ST81, ST179, and ST2181, whereas CPS5 was restricted to ST387, ST506 and ST1886.

In *E. faecium*, 5 STs were identified with ST17 being the predominant type (9/18, 50.0%), followed by ST262 (22.2%), ST117 (16.7%), ST267 (5.6%), and the novel ST3096 (5.6%). All STs belonged to clonal complex 17 (CC17). ST3096 (isolate E15) was identified as a single-locus variant of ST117 due to a novel allele in the *gdh* gene and as a double-locus variant of ST17. No CPS genotype was detected in *E. faecium*.

The genome assemblies and corresponding MLST profiles of *E. faecalis* isolates E11 and E38 and *E. faecium* isolate E15 were submitted to the PubMLST database for assignment of novel STs, with E11 and E15 accessed on 16 April 2026 and E38 on 14 April 2026.

### 2.7. Distribution of AMR Determinants Between E. faecalis and E. faecium

**Figure 1:**
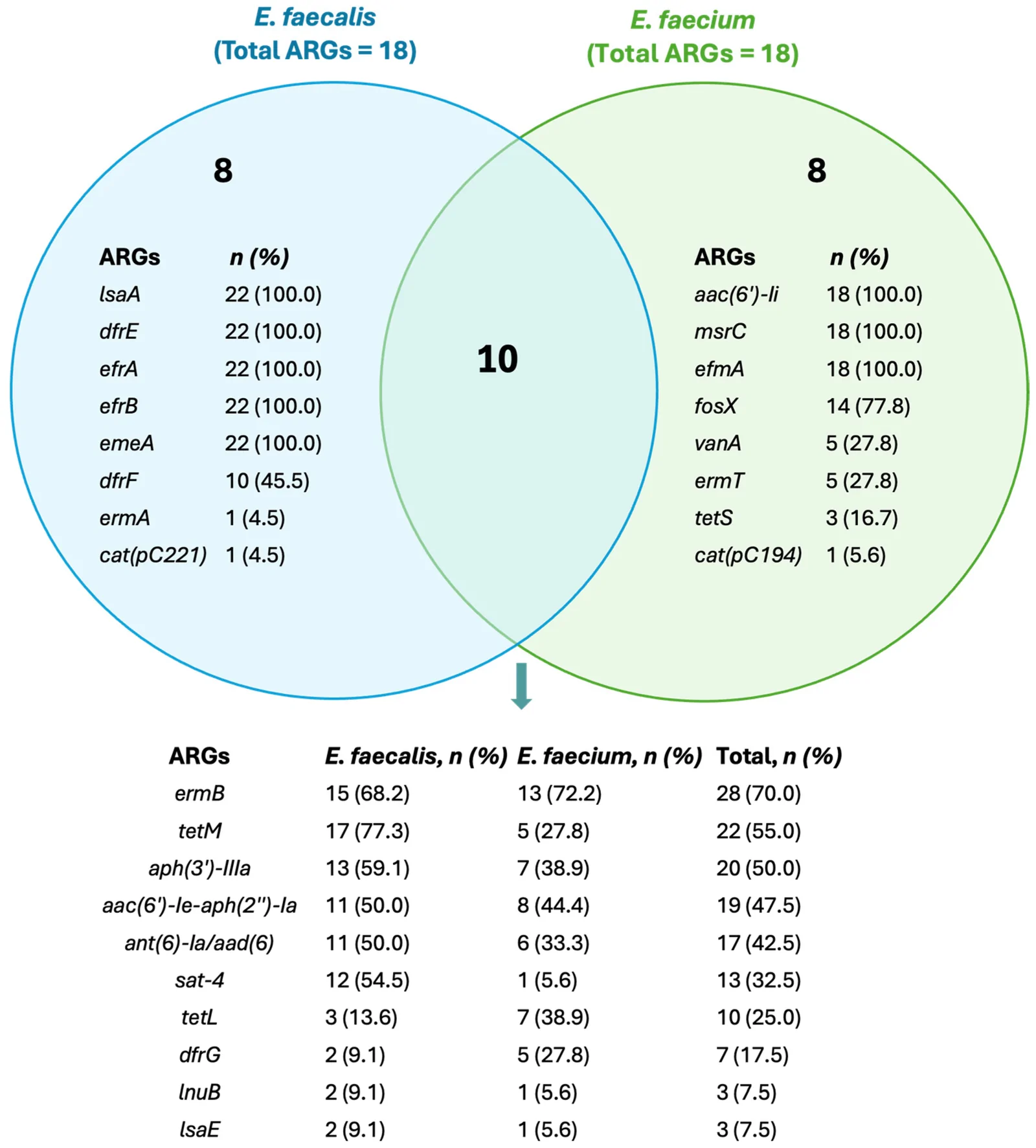
Distribution of AMR determinants among *E. faecalis* and *E. faecium* isolates.

A total of 26 AMR determinants were identified among the 40 clinical *Enterococcus* isolates, comprising 8 intrinsic chromosomal resistance determinants and 18 acquired antimicrobial resistance genes (ARGs). Ten AMR determinants were shared between the two species, whereas eight were unique to each species (Figure 1). The intrinsic resistance profile was species-specific, with *dfrE*, *lsaA*, *efrA*, *efrB*, and *emeA* detected in all *E. faecalis* isolates, whereas *aac(6*′*)-Ii*, *msrC*, and *efmA* were detected in all *E. faecium* isolates. The remaining 18 determinants were classified as acquired ARGs including *vanA*, *aac(6*′*)-Ie-aph(2*″*)-Ia*, *ant(6)-Ia/aad(6)*, *aph(3*′*)-IIIa*, *fosX*, *lnuB*, *ermA*, *ermB*, *ermT*, *lsaE*, *tetL*, *tetM*, *tetS*, *cat(pC221*), *cat(pC194)*, *sat-4*, *dfrF*, and *dfrG*.

Among the 10 acquired ARGs shared between species, *ermB* was the most prevalent (70.0%), followed by *tetM* (55.0%) and *aph(3*′*)-IIIa* (50.0%), *aac(6*′*)-Ie-aph(2*″*)-Ia* (47.5%), *ant(6)-Ia* (42.5%) and *sat-4* (32.5%). The remaining four genes including *tetL*, *dfrG*, *lnuB*, and *lsaE*, were detected at frequencies below 30%. Notably, *tetM*, *sat-4*, and aminoglycoside resistance genes (*aph(3*′*)-IIIa*, *aac(6*′*)-Ie-aph(2*″*)-Ia*, and *ant(6)-Ia/aad(6)*) were more frequently identified in *E. faecalis*, whereas *tetL* and *dfrG* were more prevalent in *E. faecium*.

### 2.8. AMR Determinant Profiles Across STs and Genotype–Phenotype Concordance

**Figure 2:**
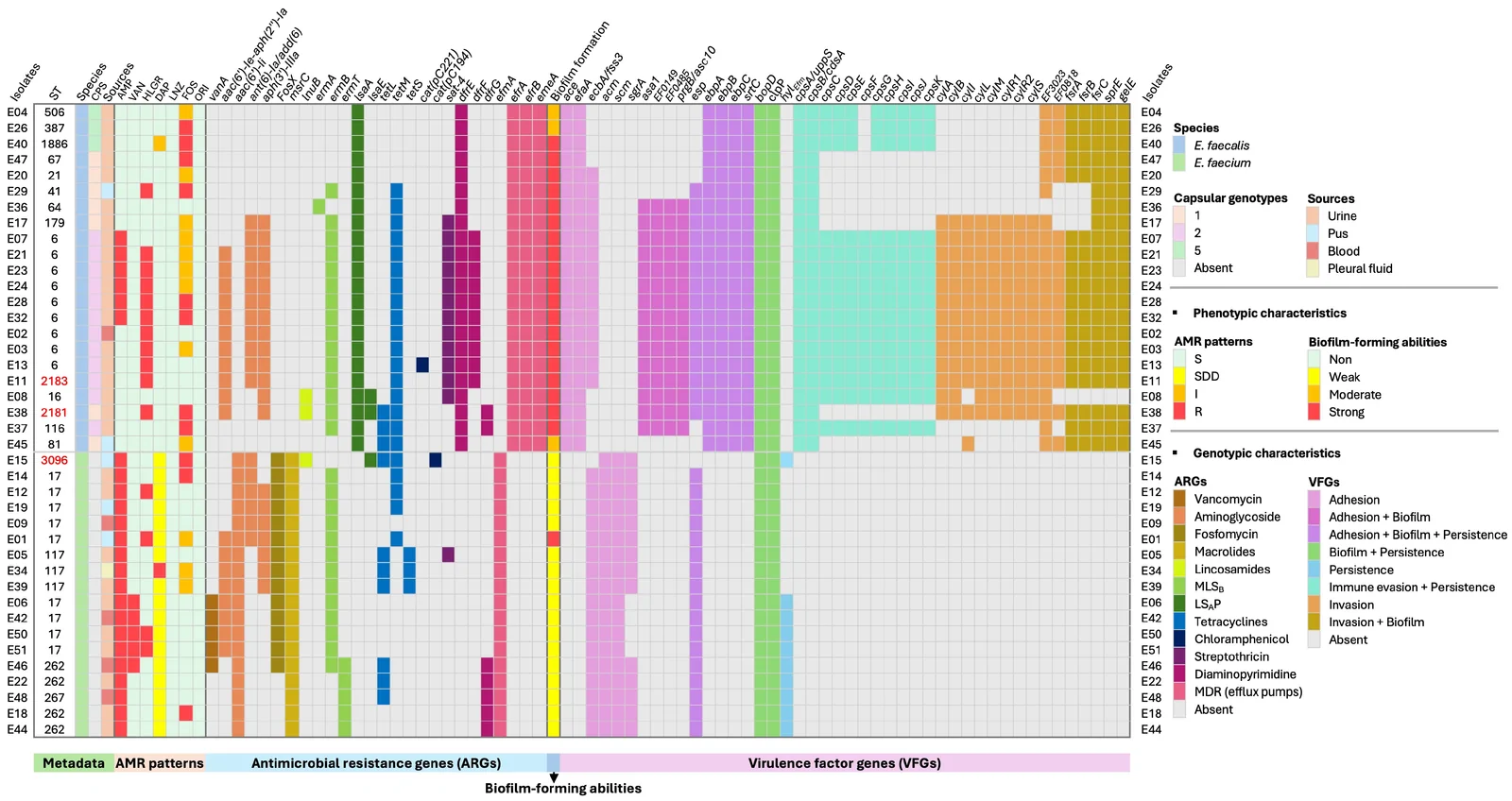
Distribution of AMR patterns, biofilm-forming abilities, ARGs, and VFGs among *E. faecalis* and *E. faecium* isolates. Novel STs are highlighted in red. MLS_B_ refers to macrolides, lincosamides, and streptogramin B antibiotics, whereas LS_A_P refers to lincosamides, streptogramin A, and pleuromutilin antibiotics.

The distribution of ARGs varied among STs of both *E. faecalis* and *E. faecium* (Figure 2). Among the 14 *E. faecalis* STs, 11 distinct ARG profiles were identified, comprising 5–13 ARGs per profile. The predominant ST6 lineage exhibited relatively conserved profiles, harboring 11–13 ARGs. Most ST6 isolates carried *lsaA*, *dfrE*, *efrA*, *efrB*, *emeA*, *ermB*, *tetM*, *sat-4*, and *dfrF*, together with varying combinations of *aac(6*′*)-Ie-aph(2*″*)-Ia* and *cat(pC221)*. Several of these determinants were also detected in other lineages, particularly ST16, ST2181, and ST2183, indicating their broad distribution among *E. faecalis* isolates.

Among the five *E. faecium* STs, 11 distinct ARG profiles were identified, comprising 5–9 ARGs per profile. The predominant ST17 lineage commonly carried *aac(6*′*)-Ii*, *msrC*, *efmA*, and *ermB*, together with varying combinations of *vanA*, aminoglycoside resistance genes, and *tetM*. Notably, *tetM* was detected in 4/5 non-VRE ST17 isolates but was absent from VRE ST17 isolates. ST262 and ST267 showed related ARG profiles but differed in the presence of *vanA*, *ermB*, and *tetL*. Interestingly, the novel ST2181 (*E. faecalis*) and ST3096 (*E. faecium*) exhibited unique ARG profiles comprising 13 and 9 ARGs, respectively. ST2181 shared several determinants with other *E. faecalis* lineages. ST3096 harbored the *lnuB-lsaE* genes, which were also detected among *E. faecalis* isolates. Moreover, *cat(pC194)* was detected exclusively in ST3096 within this isolate collection.

Genotype–phenotype concordance varied among resistance phenotypes (Figure 2). All five VRE isolates were *E. faecium*, comprising four ST17 and one ST262, and all carried *vanA* within the *vanHAX* operon. Among 11 HLGR *E. faecalis*, eight belonged to ST6 and one each to ST41, ST2181, and ST2183. The *aac(6*′*)-Ie-aph(2*″*)-Ia* gene was detected in all HLGR ST6, ST2181, and ST2183 isolates but was absent from ST41. All four HLGR *E. faecium* belonged to ST17, three of which carried *aac(6*′*)-Ie-aph(2*″*)-Ia*. Overall, this gene was detected in 13/15 HLGR isolates (86.7%) across both species. However, six of the 19 gene-positive isolates (31.6%) did not exhibit HLGR, five of which (83.3%) were *E. faecium*, indicating incomplete genotype–phenotype concordance. In addition, co-resistance to HLGR and AMP was observed in five *E. faecalis* isolates (22.7%; all ST6) and four *E. faecium* isolates (22.2%; all ST17), with the latter exhibiting high-level AMP resistance. Both predominant lineages also harbored multiple resistance determinants, highlighting their MDR characteristics.

For AMP resistance, genotype–phenotype concordance differed between species (Table 6). In *E. faecalis*, a PBP4 mutation profile including T418A and L475M was detected in 10 isolates (nine ST6 and one ST2183), of which six (60.0%, all ST6) were AMP resistant. In contrast, all high-level AMP-resistant *E. faecium* isolates harbored multiple mutations in the C-terminal region of PBP5, including the S466′ insertion and several amino acid substitutions. Notably, M485A, N496K, E525D, and E629V were consistently detected in these isolates. For DAP, LiaFSR mutation profiles showed incomplete concordance with phenotypic susceptibility (Table 6). The LiaS T120A and LiaR W73C substitutions were identified in all ST117 isolates and the novel ST3096 isolate. Among the four isolates harboring both substitutions, one ST117 isolate (25.0%) was DAP-resistant, while two isolates (ST117 and ST3096) showed reduced DAP susceptibility (MIC, 4 µg/mL). Notably, *E. faecalis* ST1886 exhibited an intermediate DAP phenotype and harbored the LiaS S260G substitution.

In contrast, poor genotype–phenotype concordance was observed for FOS. All FOS-intermediate and -resistant *E. faecalis* isolates lacked detectable *fos* genes and MurA mutations, except for isolate E29, which harbored the V356I substitution in MurA. Similarly, no MurA mutations were detected in all *E. faecium* isolates. Although *fosX* was commonly detected in *E. faecium* (77.8%), four isolates lacked *fosX*. Notably, one FOS-resistant isolate (E18) lacked *fosX*, whereas nine *fosX*-positive isolates remained susceptible to FOS. Thus, the presence of known *fos* genes alone did not reliably correspond to phenotypic FOS resistance.

### 2.9. Distribution of VFGs

Distinct species-specific patterns in VFG distribution between *E. faecalis* and *E. faecium* are shown in Figure 2. *E. faecalis* carried a wider variety of VFGs than *E. faecium*. Of the 44 VFGs identified, 40 were detected in *E. faecalis* and eight in *E. faecium*. Thirty-six VFGs were specific to *E. faecalis*, whereas four were exclusively detected in *E. faecium*. Four VFGs (*bopD*, *clpP*, *esp*, and *ecbA/fss3*) were shared between the two species. *bopD* and *clpP* were detected in all isolates (100%), while *esp* was the most prevalent adhesion-associated determinant, occurring in 80.0% (32/40) of all isolates, followed by *ecbA/fss3* at 77.5% (31/40).

All *E. faecalis* isolates (100%) carried multiple adhesion- and biofilm-associated genes, including *ace*, *efaA*, *ebpABC*, *srtC*, *bopD*, and *clpP*, as well as the capsular genes *cpsA* and *cpsB*. High prevalences were also observed for *EF3023 (hylA)*, *fsrC*, *gelE*, and *sprE* (95.5%; 21/22 each), *fsrA* and *fsrB* (81.8%), and *EF0818* (*hylB*) (77.3%; 17/22). The conjugative plasmid-associated VFGs *asa1*, *EF0149*, *EF0485*, and *prgB/asc10* were each detected in 68.2% (15/22) of isolates. The complete cytolysin operon (*cylABILMR1R2S*) was present in 59.1% (13/22) of *E. faecalis* isolates, most of which belonged to CPS2, particularly ST6. In addition, the capsular polysaccharide biosynthesis genes *cpsC–K* were detected in 54.5–68.2% of isolates, with *cpsF* showing the lowest prevalence (54.5%; 12/22).

In contrast, *E. faecium* possessed a more restricted VFG profile, dominated by genes involved in adhesion and host colonization. All *E. faecium* isolates carried *acm*, *scm*, *bopD*, and *clpP* (100%), while *esp* and *ecbA/fss3* were detected in 94.4% (17/18) of isolates and *sgrA* in 77.8% (14/18). Notably, *sgrA* was absent from all VRE ST17 isolates, whereas *ecbA/fss3* and *esp* were absent from the novel ST3096 isolate. The species-specific VFG *hylEfm* was detected in 55.6% (10/18) of *E. faecium* isolates, including all VRE isolates (5/5), as well as isolates belonging to ST262, ST267, and the novel ST3096.

Interestingly, strong biofilm production was predominantly associated with *E. faecalis* CPS2 (100%; 12/12) and CPS1 (85.7%; 6/7), whereas most CPS5 isolates were moderate biofilm producers (66.7%; 2/3). Overall, the predominant ST6 lineage (CPS2) harbored a broad range of ARGs and VFGs, with 5/9 isolates (55.6%) displaying an MDR phenotype and all exhibiting strong biofilm-forming ability. Among *E. faecium*, only one ST17 isolate was a strong biofilm producer, while all remaining isolates exhibited weak biofilm production. However, no clear concordance between biofilm-forming ability and the distribution of VFGs could be established among both species.

### 2.10. Phylogenetic Relationships and Genetic Diversity Among Enterococcus spp. Isolates

**Figure 3:**
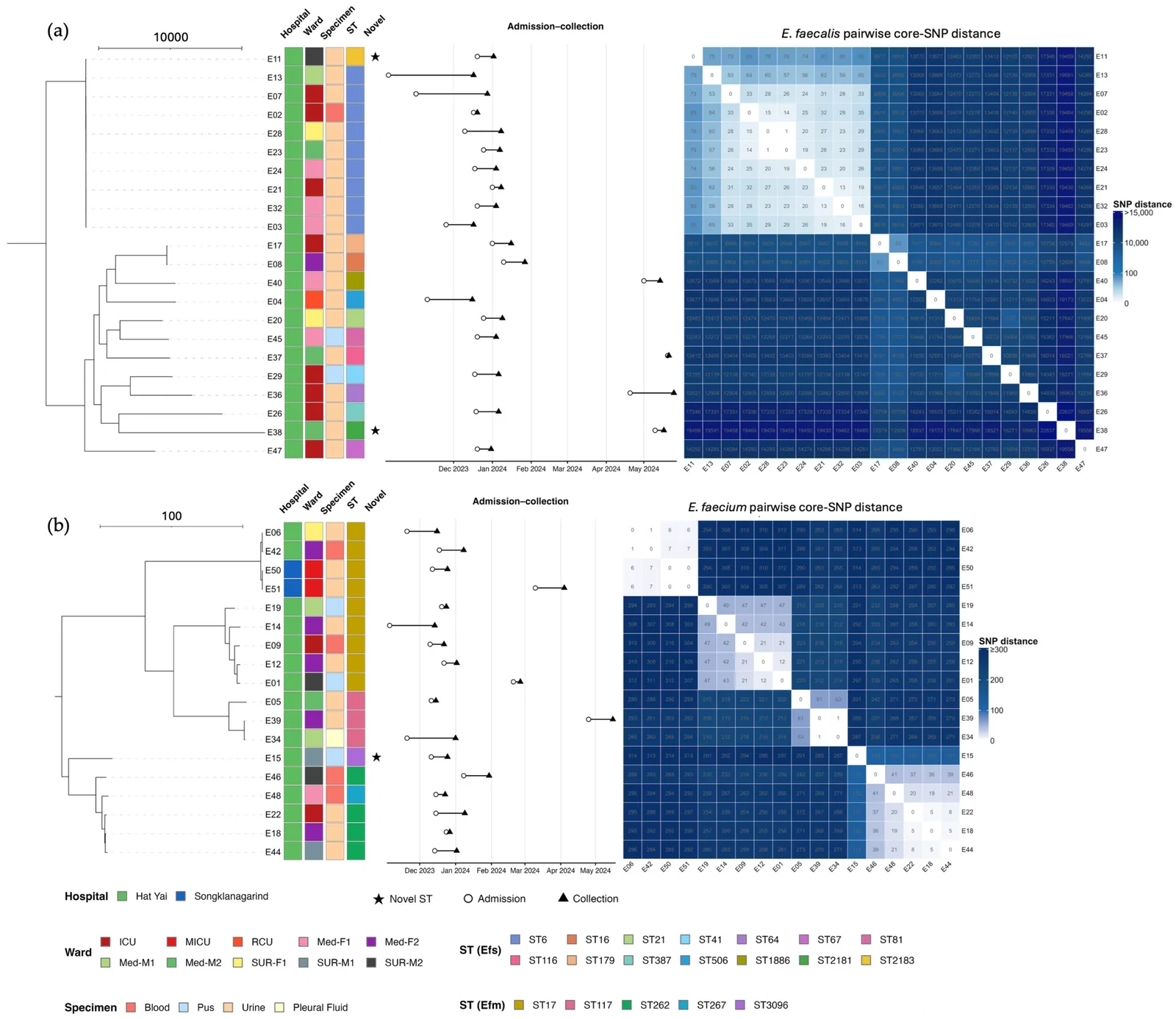
Integrated cgSNP phylogeny, epidemiological metadata, hospitalization timelines, and pairwise cgSNP distances for 22 *E. faecalis* (**a**) and 18 *E. faecium* (**b**) clinical isolates. Maximum-likelihood phylogenies were reconstructed from recombination-filtered cgSNP alignments. Metadata columns indicate hospital, ward, specimen source, and ST. Novel STs are indicated by a star (★). The hospitalization timeline displays the duration of hospitalization between admission (○) and specimen collection (▲). Pairwise cgSNP distance heatmaps are shown on the right, with isolates ordered according to the phylogenetic tree. Lower cgSNP distances (lighter colors) indicate closer genomic relatedness, whereas higher cgSNP distances (darker colors) represent greater genetic divergence.

cgSNP phylogenetic analysis revealed greater genetic diversity among *E. faecalis* than *E. faecium* isolates, characterized by a larger number of distinct lineages and substantially greater pairwise cgSNP distances (Figure 3). Among the 22 *E. faecalis* isolates, two closely related clusters (< 100 cgSNPs) were identified, comprising ST6/ST2183 and ST16/ST179, whereas the remaining isolates formed 10 genetically distinct singletons, with pairwise distances exceeding 7000 cgSNPs and reaching > 22,000 cgSNPs. The nine ST6 isolates formed a relatively closely related cluster (1–65 cgSNPs) and the smallest distance observed between E28 and E23 (1 cgSNP). Despite representing a novel ST, ST2183 was closely related to the ST6 cluster, differing by only 73–86 SNPs. Conversely, a novel ST2181 was the most genetically divergent *E. faecalis* lineage, differing from the other isolates by approximately 12,000 to > 22,000 cgSNPs.

In contrast, 18 *E. faecium* isolates exhibited several clearly separated genomic groups, with very low pairwise cgSNP distances ranging from 1 to 314 SNPs. The isolates were distributed among four major genomic clusters, comprising VRE ST17, non-VRE ST17, ST117, and ST262/ST267, while a novel ST3096 represented a distinct lineage. Although ST3096 was the most genetically divergent *E. faecium* isolate overall, it was most closely related to the VRE ST262 (E46), differing by 119 cgSNPs. Interestingly, the ST17 isolates were clearly separated into two genomic lineages associated with VRE status, comprising a highly homogeneous VRE cluster (E06, E42, E50, and E51; 0–7 cgSNPs) and a more diverse non-VRE cluster (E19, E14, E09, E12, and E01; 12–49 cgSNPs). Distances between the VRE and non-VRE ST17 clusters were substantially greater with 290–312 cgSNPs. Moreover, the non-VRE ST17 cluster was phylogenetically closer to the ST117 cluster than to the VRE ST17 cluster. Thus, despite sharing the same MLST designation, the ST17 isolates exhibited substantial core-genome diversity, with the VRE isolates forming a distinct and highly conserved genomic lineage.

### 2.11. Genomic Epidemiology and Spatiotemporal Patterns

Integration of spatiotemporal data with cgSNP analysis identified several groups of closely related isolates, with some showing patterns consistent with potential epidemiological linkage or lineage persistence (Figure 3). Among *E. faecalis*, genomic diversity was observed within ST6 (1–65 cgSNPs). However, E23 and E28 differed by only one cgSNP, had overlapping hospitalization periods in different wards, and were sampled one day apart, indicating close genomic relatedness with a possible epidemiological link.

For *E. faecium*, VRE ST17 isolates E06, E42, E50, and E51 were closely related (0–7 cgSNPs). E06 and E42 differed by one cgSNP and were identified at Hat Yai Hospital within a relatively short temporal interval, indicating close genomic relatedness with a possible epidemiological link. E50 and E51 showed no cgSNP differences and were collected from the same ward at Songklanagarind Hospital approximately three months apart, a pattern consistent with potential persistence or circulation of the VRE ST17 lineage. Although closely related VRE ST17 isolates were identified in both hospitals, limited sampling and the absence of direct epidemiological links precluded conclusions regarding inter-hospital circulation or transmission. Similarly, ST117 isolates E34 and E39 differed by only one cgSNP despite being collected approximately 4.5 months apart, a pattern consistent with potential persistence or circulation of a closely related lineage at Hat Yai Hospital. ST262 isolates E22, E18, and E44 were also closely related (5–8 cgSNPs) and had overlapping hospitalization periods across different wards at Hat Yai Hospital, a pattern consistent with a potential epidemiological link. Collectively, these findings demonstrate close genomic relatedness among several *E. faecium* lineages, with spatiotemporal patterns that may reflect epidemiological linkage or lineage persistence.

## 3. Discussion

This study provides an integrated phenotypic and genomic characterization of clinical *E. faecalis* and *E. faecium* isolates from two tertiary hospitals in southern Thailand. In contrast to most Thai studies over the past decade, which have predominantly focused on VREfm [17,18,19,20,21,22], the present study included both *E. faecalis* and *E. faecium* irrespective of VAN susceptibility and integrated antimicrobial susceptibility testing, biofilm phenotyping, WGS-based ARG and VFG profiling, MLST, and cgSNP analysis incorporating spatiotemporal clinical metadata. This comprehensive analysis expands our understanding of the resistance, virulence, and genomic epidemiology of clinical enterococci circulating in southern Thailand.

In this study, most isolates (97.5%) were collected > 48 h after hospital admission, with urinary tract specimens being the most common source (72.5%) for both *E. faecalis* and *E. faecium*. Although both species are recognized healthcare-associated pathogens [23], the timing of specimen collection alone is insufficient to distinguish nosocomial acquisition from pre-existing colonization or infection. Urinary specimens represented an important source of enterococcal isolates in the present study, with *E. faecalis* (86.4%) more frequently recovered than *E. faecium* (55.6%). A similar pattern was observed in a recent UTI cohort from a Thai tertiary hospital, in which *E. faecalis* was the third most frequently recovered organism from urine cultures and was substantially more common than *E. faecium* [24]. In contrast, urinary specimens have been a prominent source of VRE in Thai healthcare settings, accounting for 28.9% of VRE isolates in southern Thailand [17], 75.0% of VREfm isolates in Bangkok [25], and 88.4% of clinical VREfm isolates in northern Thailand [22]. Collectively, these findings highlight the urinary tract as an important clinical source of both major *Enterococcus* species in Thailand, with VREfm representing a particular concern among hospitalized patients.

VRE was observed in 12.5% (5/40) of enterococcal isolates in our study, compared with a VRE prevalence of 9.6% previously reported among nosocomial enterococcal infections in southern Thailand [17]. VRE was confined to *E. faecium* in our collection (27.8%, 5/18), all of which carried *vanA*. This rate was similar to the 36.7% VAN resistance reported among clinical *E. faecium* isolates in Bangkok [25], while the universal presence of *vanA* among our VREfm isolates was consistent with previous VREfm collections across Thailand [17,20,22,25]. Globally, VAN non-susceptibility among *E. faecium* varies considerably by geographic region, with a median rate of 21.8% across 66 countries [26]. Resistance is predominantly mediated by acquired *vanA* and *vanB* gene clusters, which remodel peptidoglycan precursors from D-Ala–D-Ala to D-Ala–D-Lac, markedly reducing VAN binding [27]. The *vanA* cluster, commonly associated with the Tn1546 transposon and transferable plasmids, typically confers high-level resistance to both VAN and teicoplanin [27].

Distinct AMP resistance profiles were observed between *E. faecalis* and *E. faecium* in our study. All *E. faecium* isolates exhibited high-level AMP resistance, compared with 27.3% of *E. faecalis* isolates, which had comparatively lower MICs. Global surveillance data from 2019–2023 showed a similar species-specific pattern, with AMP resistance reported in 85.7% of *E. faecium* and only 0.4% of *E. faecalis* isolates [8]. The higher rates observed in our study may reflect its small sample size and hospital-specific population. Similarly, 100% AMP resistance among *E. faecium* isolates has been reported in recent Thai studies [18,22,25]. However, PBP5 amino acid alterations associated with AMP resistance have not been characterized in Thai clinical *E. faecium*. High-level AMP resistance in *E. faecium* is primarily associated with alterations in the low-affinity PBP5, particularly within its C-terminal transpeptidase domain [28,29]. Several PBP5 alterations identified in our isolates, including the S466′ insertion, M485A, and E629V, have been associated with increased AMP resistance, particularly when occurring in combination [30,31]. Among *E. faecalis*, six of 10 isolates carrying PBP4 substitutions were AMP resistant. A369V, T418A, L475M, A536T, and D666P have previously been reported in isolates with reduced β-lactam susceptibility [32,33] whereas, to our knowledge, A464S has not been reported in *E. faecalis* PBP4. Its co-occurrence with these alterations may represent a distinctive PBP4 profile among ST6/ST2183 isolates, although its contribution to AMP resistance remains unclear.

HLGR prevalence among enterococci shows considerable geographic and population-level variation. In the present study, HLGR was more frequent in *E. faecalis* (50.0%) than in *E. faecium* (22.2%), with *aac(6*′*)-Ie-aph(2*″*)-Ia* detected in 86.7% (13/15) of HLGR isolates. Lower rates were reported among urinary *E. faecalis* isolates in Iran (18.8%) [34] and among invasive *E. faecalis* and *E. faecium* isolates in Japan (34% and 9%, respectively), where all HLGR isolates carried *aac(6*′*)-Ie-aph(2*″*)-Ia* [35]. In contrast, a recent Chinese bloodstream infection study reported rates of 51.9% and 54.2% in *E. faecalis* and *E. faecium*, respectively [36]. In Thailand, contemporary HLGR data for clinical *E. faecalis* remain limited, while rates of 9.3% and 14.6% have been reported among clinical and surveillance VREfm isolates, respectively, in northern Thailand [22] compared with no HLGR among 52 clinical VREfm isolates from Bangkok [25]. Although *aac(6*′*)-Ie-aph(2*″*)-Ia* is a major determinant of HLGR [5], 6 of 19 gene-positive isolates (31.6%) in our study lacked the HLGR phenotype, 5 of which were *E. faecium*. Similar genotype–phenotype discordance, predominantly involving *E. faecium*, has been reported and may result from disruption of *aac(6*′*)-Ie-aph(2*″*)-Ia* by IS1216V [37]. In addition, four *E. faecium* ST17 isolates, two of which were VRE, exhibited both HLGR and high-level AMP resistance. This co-resistance profile may limit the utility of conventional β-lactam–aminoglycoside combination therapy, as HLGR can abolish the synergistic bactericidal activity of GEN with cell-wall-active agents [38].

FOS is of particular interest because susceptibility data for clinical enterococci in Thailand remain limited. Resistance was higher in *E. faecalis* than in *E. faecium* (36.4% vs. 16.7%), contrasting with previous reports of greater resistance in *E. faecium* [39,40]. The resistance rate in *E. faecalis* was also considerably higher than the 8.1% (*n* = 3200) and 7.3% (*n* = 3733) reported among urinary isolates in Thailand in 2017 and 2018, respectively [41]. Similarly, a Chinese multicenter study reported a low FOS resistance rate (3.5%), predominantly in *E. faecium*, with frequent detection of *fosX* and *fosB* [42]. MurA alterations have also been implicated in FOS resistance in enterococci [39]. In our study, a MurA V356I substitution was identified in one FOS-resistant *E. faecalis* isolate; however, its role in FOS resistance remains unclear. Notably, FOS non-susceptibility was not consistently associated with the detected genetic determinants. Similar genotype–phenotype discordance has been reported previously, with *fosX* detected in both FOS-susceptible and -resistant enterococci [39]. This discordance may partly reflect differences in gene expression, as increased *fosX* expression has been associated with higher levels of FOS resistance [39,42]. In addition, FOS-resistant *E. faecium* isolates lacking known *fos* resistance genes and harboring wild-type *murA* have also been reported [43]. More recently, Peng et al. found no specific association between detected MurA substitutions and FOS resistance, while *fosA*, *fosB*, *fosC*, and *fosX* were absent from the FOS-resistant or intermediate isolates examined [44]. Collectively, these findings indicate that the genetic basis of FOS non-susceptibility in enterococci remains incompletely understood and may involve mechanisms beyond the presence of currently recognized resistance determinants.

DAP remains an important therapeutic option for MDR enterococcal infections, particularly VREfm. DAP resistance in *E. faecium* varies geographically, with global rates ranging from 3.3% to 13.4% [8]. In the present study, the DAP-resistant *E. faecium* isolate (MIC, 8 µg/mL) carried the LiaS T120A and LiaR W73C substitutions. These co-occurring substitutions are associated with reduced DAP susceptibility and resistance in *E. faecium* and act epistatically to enhance LiaFSR signaling in response to DAP-induced membrane stress [11,45]. However, they do not invariably predict phenotypic resistance, as they were detected in one isolate with low DAP MIC (0.5 µg/mL) in our study and have previously been reported in DAP-SDD *E. faecium* [45]. Notably, one DAP-intermediate *E. faecalis* isolate harbored LiaS S260G. Although its significance remains unknown, a nearby LiaS H264Q substitution has been reported within the predicted ATP-binding domain in an *E. faecium* isolate with reduced DAP susceptibility [46], warranting further investigation of S260G. In contrast to our findings, previous Thai studies generally reported preserved DAP activity, with no resistant VREfm detected [18,47]. Differences in study populations, hospital settings, antimicrobial exposure, or circulating lineages may partly explain this variation.

LZD and ORI remained highly active in vitro, with all isolates in this study susceptible to both agents. Previous surveillance studies have demonstrated high activity of LZD and ORI against *Enterococcus* spp., including VRE and other resistant subsets [48,49]. Similarly, recent Thai studies reported no LZD resistance among VREfm isolates from northeastern Thailand [18], regional tertiary hospitals including those in southern Thailand [20], and Bangkok [25]. These findings support the continued high in vitro activity of LZD against clinical VREfm in Thailand. However, published susceptibility data for ORI among clinical enterococci in Thailand appear to be lacking, precluding direct comparison with previous Thai studies.

In the present study, biofilm-forming ability differed substantially between species (*p* < 0.001), with 86.4% of *E. faecalis* exhibiting strong biofilm formation, compared with predominantly weak biofilm formation among *E. faecium* (94.4%). This species-specific difference is in line with previous reports of greater biofilm-forming capacity in *E. faecalis* [14,50]. Thai studies have reported variable biofilm phenotypes among VREfm [20,22,51]. These differences may partly reflect assay conditions; our study used TSB with 1% glucose, whereas Nokkhiao et al., 2025 [22], who reported predominantly moderate biofilm formation, used BHI with 2% glucose. Notably, BHI with 2% glucose has been shown to promote greater biofilm formation than TSB with 1% glucose in both species, particularly *E. faecium* [52], highlighting the need for caution when comparing studies.

*E. faecalis* exhibited a greater diversity of VFGs than *E. faecium* (40 vs. 8), although VFG distribution did not clearly correspond to biofilm strength, supporting the multifactorial nature of biofilm formation [53,54]. Notably, all nine ST6 isolates were strong biofilm producers and five (55.6%) were MDR. Although strong biofilm formation has not been clearly established as a lineage-specific characteristic of ST6, MDR has been reported among clinical ST6/CC2 isolates [55]. Strong biofilm production predominated among CPS2 (100%) and CPS1 (85.7%) isolates, whereas CPS5 isolates were mainly moderate producers. However, the small sample size and limited supporting evidence preclude firm conclusions regarding an association between CPS genotype and biofilm formation, warranting further investigation in larger isolate collections.

In this study, ST6 was predominant among *E. faecalis* (40.9%). Although comparable MLST data for clinical *E. faecalis* in Thailand remain limited, substantial lineage diversity has been reported in neighboring Malaysia, including ST6 among clinical isolates [56]. Notably, two closely related ST6 isolates in our study differed by only one cgSNP and had overlapping hospitalization periods in different wards, indicating a possible epidemiological link. Nosocomial dissemination of ST6 has previously been documented in a prolonged outbreak of VREfs across two Spanish hospitals [57]. ST6/CC2 has also been frequently identified among hospitalized patients [55], while large-scale genomic analysis has identified ST6 as a major lineage within a hospital-associated *E. faecalis* cluster [58].

Among *E. faecium*, ST17 was predominant (50.0%), with closely related isolates within VRE ST17, ST262, and ST117 showing notable spatiotemporal patterns. In Thailand, ST17 predominated among invasive and hospital-associated VREfm populations (72.4–86.5%), while ST117 and ST262 were detected at lower frequencies in separate hospital cohorts [19,25]. The close genomic relatedness and spatiotemporal patterns observed among these lineages may reflect epidemiological linkage or lineage persistence within healthcare settings. Notably, all three lineages have previously been implicated in nosocomial VREfm outbreaks. ST17 caused a clonal MDR-VREfm outbreak in a Spanish haemo-oncology ward [59], ST117 has been associated with hospital outbreaks in Europe [60], and ST262 was the dominant clone in a VREfm outbreak involving 13 patients in a UK hematology ward [61]. These findings, together with the documented outbreak potential of these lineages, support continued genomic surveillance in healthcare settings. Nevertheless, these epidemiological implications should be interpreted cautiously. Genomic relatedness and spatiotemporal proximity alone are insufficient to establish direct transmission without supporting patient-level epidemiological evidence.

The identification of novel STs further highlights the genomic diversity of clinical enterococci in our setting. The novel *E. faecalis* ST2183 was phylogenetically related to the predominant ST6 lineage, whereas ST2181 and the novel *E. faecium* ST3096 were more genetically distinct. All carried multiple acquired ARGs. Although ST2183 and ST3096 were defined by allelic variations in *gki* and *gdh*, respectively, these variations have no established role in AMR. Their AMR phenotypes are more appropriately interpreted in relation to acquired ARGs and resistance-associated chromosomal mutations. ST3096 was particularly notable for its AMR phenotype, characterized by high-level AMP resistance, FOS resistance, and reduced DAP susceptibility (MIC, 4 µg/mL). Consistent with its reduced DAP susceptibility, ST3096 also harbored co-occurring LiaS T120A and LiaR W73C substitutions associated with elevated DAP MICs [11,45]. The emergence of this AMR profile in a novel genetic background warrants further surveillance.

Several limitations should be considered when interpreting the findings of the present study. Given the relatively small sample size and uneven sampling between the two hospitals, this study should be considered a targeted, high-resolution genomic surveillance study rather than a prevalence survey. This limitation is particularly relevant to the relatively high resistance rates observed for several antibiotics. Therefore, observed phenotype and genotype proportions should not be interpreted as representative prevalence estimates or directly compared with larger surveillance studies. Moreover, the cross-sectional sampling design and limited epidemiological data restricted our ability to reconstruct potential transmission pathways. WGS-based detection of resistance-associated mutations, ARGs, and virulence-associated genes should be interpreted with caution. Their presence does not establish causality or confirm gene expression or functional activity, particularly when genotype–phenotype concordance is incomplete. Mutation analysis was restricted to selected AMP-, DAP-, and FOS-associated targets, potentially leaving other resistance mechanisms undetected. Further functional and gene-expression studies are needed to clarify the contributions of these genetic determinants to the observed phenotypes. In addition, although short-read sequencing was sufficient for high-resolution cgSNP clustering and ARG/VFG profiling, future studies utilizing long-read sequencing will provide complete reconstruction of plasmids and other mobile genetic elements to fully trace horizontal gene transfer pathways.

## 4. Materials and Methods

### 4.1. Bacterial Isolates and Culture Conditions

A total of 40 non-duplicate clinical *Enterococcus* isolates were retrospectively collected from leftover cultures obtained during routine diagnostic laboratory procedures at Hat Yai Hospital (*n* = 38) and Songklanagarind Hospital (*n* = 2), Songkhla, southern Thailand, between December 2023 and May 2024. The isolates were recovered from four specimen types: urine, blood, pus, and pleural fluid. Species-level identification was performed using matrix-assisted laser desorption/ionization time-of-flight mass spectrometry (Bruker Daltonik GmbH, Bremen, Germany). All isolates were cultured in brain heart infusion (BHI) broth or agar (HiMedia Laboratories Pvt. Ltd., Mumbai, India) at 37 °C for 16–20 h.

### 4.2. Antimicrobial Susceptibility Testing

Antimicrobial susceptibility was determined by broth microdilution according to Clinical and Laboratory Standards Institute (CLSI) guidelines [62]. Seven antimicrobial agents were tested over the following concentration ranges (µg/mL): AMP (0.25–128), VAN (0.25–128), GEN (2–1024, DAP (0.06–32), LZD (0.25–32), FOS (4–2048), and ORI (0.001–0.48). Minimum bactericidal concentration (MBC) was defined as the lowest concentration of an antibacterial agent capable of killing the tested bacteria. *Staphylococcus aureus* ATCC 29213 and *E. faecalis* ATCC 29212 were used as quality-control strains. Each experiment was performed in triplicate.

MICs were interpreted according to CLSI breakpoints [62]. Isolates with AMP MICs ≥ 64 µg/mL were classified as exhibiting high-level AMP resistance. Isolates with GEN MICs > 500 µg/mL were classified as HLGR, whereas those with MICs ≤ 500 µg/mL were classified as non-HLGR. For DAP, species-specific MIC breakpoints were applied: *E. faecium* isolates were classified as SDD at MICs ≤ 4 µg/mL and resistant at ≥8 µg/mL, whereas *E. faecalis* isolates were classified as susceptible at MICs ≤ 2 µg/mL, intermediate at 4 µg/mL, and resistant at ≥8 µg/mL. For FOS, MICs were categorized using the CLSI urinary *E. faecalis* breakpoints (susceptible, ≤64 µg/mL; intermediate, 128 µg/mL; resistant, ≥256 µg/mL) [62]. In the absence of established breakpoints for *E. faecium*, these criteria were also applied for descriptive purposes, consistent with a previous study [42]. In addition, ORI MICs were interpreted with caution. CLSI defines a susceptible breakpoint of ORI (≤0.12 µg/mL) only for VAN-susceptible *E. faecalis*. Because no categorical breakpoint is available for other enterococci, this threshold was applied descriptively to all isolates, as previously used in surveillance studies [49].

For AMR pattern analysis, isolates categorized as intermediate were considered non-susceptible. *Enterococcus* spp. isolates resistant or non-susceptible to three or more antimicrobial agents from different classes were classified as MDR.

### 4.3. Screening of Biofilm-Forming Ability

Biofilm-forming ability was evaluated using a crystal violet microtiter plate assay modified from previously described methods [14,52]. Fresh colonies from 18–24-h agar cultures were selected, and three to five colonies with identical morphology were suspended in sterile normal saline. The bacterial suspension was adjusted to a 0.5 McFarland standard. Twenty microliters of the bacterial suspension were added to 180 µL of tryptic soy broth (TSB) supplemented with 1% glucose in sterile flat-bottomed 96-well polystyrene microtiter plates. The plates were incubated statically at 37 °C for 24 h. After incubation, the culture medium was carefully removed, and each well was gently washed three times with 200 µL of 1× sterile phosphate-buffered saline (PBS). The adherent biofilms were fixed with 200 µL of anhydrous ethanol for 20 min and allowed to air-dry. The wells were stained with 200 µL of 0.1% crystal violet for 15 min, washed three times with sterile distilled water, and air-dried. The bound crystal violet was solubilized with 200 µL of 95% ethanol for 30 min. The optical density was measured at 570 nm using a microplate reader. Sterile TSB supplemented with 1% glucose without bacterial inoculation was used as the negative control. Each isolate was tested in quadruplicate in two independent experiments.

The optical-density cut-off (ODc) was defined as the mean OD of the negative-control wells plus three standard deviations [63]. Isolates were classified as non-biofilm producers (OD ≤ ODc), weak producers (ODc < OD ≤ 2ODc), moderate producers (2ODc < OD ≤ 4ODc), or strong producers (OD > 4ODc). Differences in biofilm formation between *E. faecalis* and *E. faecium* were assessed using the Mann–Whitney U test based on the mean OD_570_ value of each isolate. A *p*-value < 0.05 was considered statistically significant.

### 4.4. Genomic DNA Extraction and WGS

Genomic DNA was extracted using a modified bacterial DNA extraction procedure based on previously described methods [64,65]. DNA concentrations were measured using a NanoPhotometer^®^ N60 (Implen GmbH, Munich, Germany). DNA integrity and purity were verified by agarose gel electrophoresis. The purified DNA samples were submitted to the Tsingke biotechnology Co., Ltd., (Beijing, China) for short-read WGS. Libraries with a 350 bp insert size were prepared using the TruSeq™ Nano DNA Sample Prep Kit (Illumina, San Diego, CA, USA) and sequenced on NovaSeq 6000 platform (Illumina, San Diego, CA, USA) with 150 bp paired-end reads.

### 4.5. De Novo Assembly, Genome Annotation and Pan-Genome Analysis

De novo genome assembly was performed using Unicycler v0.4.8, followed by genome annotation using Prokka v1.15.6 and RASTtk [66]. The assembled genome quality was evaluated using QUAST v5.3.0. The genome completeness and contamination were estimated using CheckM v1.2.5 algorithm. Genome annotations were additionally examined using the Bacterial and Viral Bioinformatics Resource Center (BV-BRC) [67].

Pan-genome analysis was performed using Roary v3.13.0, with core genes defined as those present in ≥ 99% of isolates. SNPs were extracted from the Roary core-gene alignment using SNP-sites v0.8.2. Maximum-likelihood phylogenetic trees were inferred using IQ-TREE v3.0.1 with 1000 bootstrap replicates. Pan-genome profiles were visualized alongside the core-gene SNP phylogeny using Phandango v1.3.1.

### 4.6. Multilocus Sequence Typing (MLST) and Capsular Genotype Analysis

MLST profiles were derived from WGS assemblies using FastMLST v0.0.19, with allele identification and ST assignment based on the PubMLST database (https://pubmlst.org/). STs were assigned according to the MLST scheme based on the allelic profiles of seven housekeeping genes. The seven-locus MLST scheme for *E. faecalis* comprised *gdh*, *gyd*, *pstS*, *gki*, *aroE*, *xpt*, and *yqiL*, whereas the *E. faecium* scheme comprised *atpA*, *ddl*, *gdh*, *purK*, *gyd*, *pstS*, and *adk*. Novel alleles and allele combinations not matching existing profiles were verified and submitted to PubMLST for assignment of novel alleles and STs. Capsular genotypes were determined using NCBI pairwise alignment based on reference *cps* loci [68].

### 4.7. Detection of ARGs and VFGs

ARGs and VFGs were identified using ABRicate v1.2.0 with default parameters. ARGs were screened against the Comprehensive Antibiotic Resistance Database (CARD) and ResFinder, while VFGs were screened against the Virulence Factor Database (VFDB). All identified genes were subsequently verified against the BV-BRC database (https://www.bv-brc.org/). Hits with ≥80% sequence identity and ≥ 80% sequence coverage relative to the reference sequences were considered positive. Candidate VFG genes not detected by the initial database screening, including *hylEfm* and *bopD*, were manually examined using NCBI web-based BLASTn and/or BLASTp against curated reference sequences. For VFGs containing repetitive regions, hits with ≥80% sequence identity and ≥50% reference-sequence coverage were additionally examined manually because repetitive regions may result in fragmented assemblies and reduced alignment coverage in short-read assemblies. Accession numbers of reference sequences used for manual verification are provided in Table S3.

### 4.8. Resistance-Associated Mutation Analysis

Amino acid variations in molecular targets associated with AMP, DAP and FOS resistance were investigated. Predicted amino acid sequences of PBP4 in *E. faecalis*, PBP5 in *E. faecium*, the LiaFSR regulatory proteins (LiaF, LiaS, and LiaR) and MurA were extracted from genome assemblies and aligned with corresponding reference sequences using MAFFT v7.526. Amino acid variations in PBP4 were compared with previously reported substitutions associated with reduced β-lactam susceptibility in *E. faecalis* [32,33]. Variations in PBP5 were compared with previously characterized substitutions and insertions associated with AMP resistance in *E. faecium* [28,29,30,31]. Variations in LiaF, LiaS, and LiaR were compared with previously reported substitutions associated with reduced DAP susceptibility or resistance in *E. faecium* [11,69]. Variations in MurA were compared with previously reported substitutions associated with FOS resistance [39]. Amino acid substitutions, deletions, and insertions were identified using a custom Python (v3.13.11) script. Reference strains and protein accession numbers used for sequence alignment are provided in Table S3.

### 4.9. Comparative Genomics and Epidemiological Analysis

cgSNPs were identified from Illumina paired-end reads using Snippy v4.6.0. Recombinant regions were identified and removed using Gubbins v3.4, and pairwise cgSNP distances were calculated from the recombination-filtered alignment using SNP-dists v0.8.2. A maximum-likelihood phylogenetic tree was constructed from the recombination-filtered cgSNP alignment using IQ-TREE v3.0.1 with 1000 bootstrap replicates, followed by rerooting and visualization in the Interactive Tree of Life (iTOL) v7.6.

For genomic epidemiological analysis, cgSNP phylogeny and pairwise cgSNP distances were integrated with clinical and epidemiological metadata (hospital, ward, specimen type, ST, novel ST status, and admission-to-specimen-collection timelines). Genomic relatedness was assessed using pairwise cgSNP distances and interpreted together with available spatiotemporal epidemiological data to evaluate potential epidemiological linkage. Temporally separated closely related isolates were interpreted cautiously as potentially reflecting lineage persistence or circulation. All visualizations were generated in R (v4.5.3). Heatmaps of pairwise cgSNP distances were generated using the ComplexHeatmap package v2.26.1, with isolates arranged according to the tip order of the corresponding phylogenetic tree. Clinical and epidemiological metadata were visualized using ggplot2 v4.0.3. Figure components and legends were arranged using cowplot v1.2.0.

## 5. Conclusions

In conclusion, this study provides integrated phenotypic and genomic insights into clinical *E. faecalis* and *E. faecium* circulating in healthcare settings in southern Thailand. The two species exhibited distinct resistance, virulence, and biofilm profiles. *E. faecalis* showed greater VFG diversity and stronger biofilm formation, whereas *E. faecium* showed clinically important resistance phenotypes, including VAN and DAP resistance. Genomic epidemiological analysis identified closely related isolates within ST6, ST17, ST117, and ST262, with spatiotemporal patterns that may reflect epidemiological linkage or lineage persistence, particularly among *vanA*-positive VREfm ST17 isolates. Notably, to our knowledge, this study provides the first report in Thailand of DAP-resistant *E. faecium* carrying resistance-associated LiaFSR alterations. The identification of novel STs, particularly *E. faecium* ST3096 exhibiting high-level AMP resistance, FOS resistance, and reduced DAP susceptibility with LiaFSR alterations, further highlights local genomic diversity and the emergence of clinically important resistant genetic backgrounds. These findings expand genomic epidemiological data on clinical enterococci in Thailand and emphasize the importance of integrating phenotypic susceptibility testing, WGS, and epidemiological surveillance to monitor emerging resistance and clinically important enterococcal lineages.

## Figures and Tables

**Table 1:** Clinical sources and patient demographics of 40 *Enterococcus* spp. isolates.

Clinical Data	*E. faecalis* (*n* = 22), *n* (%)	*E. faecium* (*n* = 18), *n* (%)	Total (*n* = 40), *n* (%)
Source			
Urine	19 (86.4)	10 (55.6)	29 (72.5)
Blood	1 (4.5)	4 (22.2)	5 (12.5)
Pus	2 (9.1)	3 (16.7)	5 (12.5)
Pleural fluid	0 (0.0)	1 (5.6)	1 (2.5)
Total	22 (100.0)	18 (100.0)	40 (100.0)
Host gender			
Female	14 (63.6)	9 (50.0)	23 (57.5)
Male	8 (36.4)	9 (50.0)	17 (42.5)
Total	22 (100.0)	18 (100.0)	40 (100.0)
Host age			
Range	<1–89	1–94	<1–94
Median	59	69	65

**Table 2:** Antimicrobial susceptibility of 40 *Enterococcus* spp. isolates.

Antimicrobial Agents	Antimicrobial Susceptibility, *n* (%) ^a^								
	*E. faecalis* (*n* = 22)			*E. faecium* (*n* = 18)			Total Isolates (*n* = 40)		
	S	I	R	S	SDD/I	R	S	SDD/I	R
AMP	16 (72.7)	–	6 (27.3)	0 (0.0)	–	18 (100)	16 (40.0)	–	24 (60.0)
VAN	22 (100.0)	0 (0.0)	0 (0.0)	13 (72.2)	–/0 (0.0)	5 (27.8)	35 (87.5)	–/0 (0.0)	5 (12.5)
GEN ^b^	11 (50.0)	–	11 (50.0)	14 (77.8)	–	4 (22.2)	25 (62.5)	–	15 (37.5)
DAP	21 (95.5)	1 (4.5)	0 (0.0)	–	17 (94.4)/–	1 (5.6)	21 (52.5)	17 (42.5)/1 (2.5)	1 (2.5)
LZD	22 (100.0)	0 (0.0)	0 (0.0)	18 (100.0)	–/0 (0.0)	0 (0.0)	40 (100.0)	–/0 (0.0)	0 (0.0)
FOS	5 (22.7)	9 (40.9)	8 (36.4)	12 (66.6)	–/3 (16.7)	3 (16.7)	17 (42.5)	–/12 (30.0)	11 (27.5)
ORI	22 (100.0)	–	–	18 (100.0)	–	–	40 (100.0)	–	–

^a^ S, susceptible; SDD, susceptible-dose dependent; I, intermediate; R, resistant; –, no applicable interpretive category. The SDD breakpoint applies only to DAP in *E. faecium*. ^b^ For GEN, R refers to HLGR (MIC > 500 µg/mL) and S refers to non-HLGR (MIC ≤ 500 µg/mL).

**Table 3:** MIC_50_ and MIC_90_ values of antimicrobial agents against *Enterococcus* spp.

Bacterial Species		Antimicrobial Agents (µg/mL)						
		AMP	VAN	GEN	DAP	LZD	FOS	ORI
*E. faecalis* (*n* = 22)	MIC range	2–32	0.25–2	8–>1024	0.25–2	0.5–2	64–512	0.015–0.12
	MBC range	32–>128	16–128	128–>1024	2–>32	4–>32	512–>2048	0.03–0.48
	MIC_50_	4	1	1024	0.5	2	128	0.03
	MIC_90_	32	2	>1024	2	2	512	0.12
*E. faecium* (*n* = 18)	MIC range	>128–>128	0.25–>128	2–>1024	0.5–8	0.5–2	32–512	0.002–0.12
	MBC range	>128–>128	16–>128	128–>1024	1–32	16–>32	1024–>2048	0.015–0.12
	MIC_50_	>128	1	16	2	2	64	0.004
	MIC_90_	>128	128	>1024	4	2	512	0.015
Total (*n* = 40)	MIC range	2–>128	0.25–>128	2–>1024	0.25–8	0.5–2	32–512	0.002–0.12
	MBC range	32–>128	16–>128	128–>1024	1–>32	4–>32	512–>2048	0.015–0.48
	MIC_50_	32	1	16	1	2	128	0.015
	MIC_90_	>128	64	>1024	4	2	512	0.12

**Table 4:** AMR patterns of 40 *Enterococcus* spp. isolates.

Species	AMR Pattern *	*n* (%)
*E. faecalis* (*n* = 22)	AMP–HLGR–FOS	5 (22.7)
	AMP–FOS	1 (4.5)
	HLGR–FOS	3 (13.6)
	HLGR	3 (13.6)
	DAP–FOS	1 (4.5)
	FOS	7 (31.8)
	S	2 (9.1)
*E. faecium* (*n* = 18)	AMP–VAN–HLGR	2 (11.1)
	AMP–HLGR–FOS	1 (5.6)
	AMP–DAP–FOS	1 (5.6)
	AMP–VAN	3 (16.7)
	AMP–HLGR	1 (5.6)
	AMP–FOS	4 (22.2)
	AMP	6 (33.3)

* AMR included non-susceptible isolates (I or R). S, susceptible to all antibiotics.

**Table 5:** Biofilm-forming ability of 40 *Enterococcus* spp. isolates.

Classification	*E. faecalis* (*n* = 22), *n* (%)	*E. faecium* (*n* = 18), *n* (%)	Total (*n* = 40), *n* (%)
Strong	19 (86.4)	1 (5.6)	20 (50.0)
Moderate	3 (13.6)	0 (0.0)	3 (7.5)
Weak	0 (0.0)	17 (94.4)	17 (42.5)
Non	0 (0.0)	0 (0.0)	0 (0.0)

**Table 6:** Mutation profiles of PBP4, PBP5, LiaFSR and MurA with corresponding AMP, DAP and FOS resistance phenotypes in *E. faecalis* and *E. faecium*.

Molecular Targets	Mutation Profiles ^a^	No. of Isolates; *n* (%)	ST (*n*)	R (%)	I (%)	SDD (%)
PPB4 in *E. faecalis* (*n* = 22)	A369V, T418A, A464S, L475M, A536T, D666P	10 (45.5)	6 (9), 2183 (1)	60.0	—	—
	A369V	5 (22.7)	16, 21, 67, 506, 1886	0.0	—	—
	A369V, P520S	2 (9.1)	64, 179	0.0	—	—
	K152N, I166V, Q228R, E286D, E289K, A369V	1 (4.5)	116	0.0	—	—
	S59T, E62K, E289K, A369V, A437T	1 (4.5)	387	0.0	—	—
	T53K, E289K, A369V	1 (4.5)	81	0.0	—	—
	A369V, G509C, V582I	1 (4.5)	41	0.0	—	—
	E289K, A369V	1 (4.5)	2181	0.0	—	—
PBP5 (C-terminal; aa374—679) in *E. faecium* (*n* = 18)	Q408H, Y460F, Q461K, V462T, S466′, H470Q, M485A, N496K, A499T, E525D, N546T, A558T, G582S, E629V, K632Q, P642L, P667S	10 (55.6)	17 (5), 262 (3), 267 (1), 3096 (1)	100.0	—	—
	Q408H, Y460F, Q461K, V462T, S466′, H470Q, M485A, N496K, A499T, E525D, N546T, A558I *, G582S, E629V, K632Q, P642L, P667S	4 (22.2)	17	100.0	—	—
	Q408H, Y460F, Q461K, V462T, S466′, H470Q, M485A, N496K, A499T, E525D, N546T, A558T, A570P *, G582S, E629V, K632Q, P642L, P667S	3 (16.7)	117	100.0	—	—
	Q408Y *, Y460F, Q461K, V462T, S466′, H470Q, M485A, N496K, A499T, E525D, N546T, A558T, G582S, E629V, K632Q, P642L, P667S	1 (5.6)	262	100.0	—	—
LiaF/LiaS/LiaR in *E. faecalis* (*n* = 22)	V45I/—/V365A	1 (5.6)	64	0.0	0.0	—
	—/S260G/—	1 (5.6)	1886	0.0	100.0	—
	—/V365A/—	1 (5.6)	2181	0.0	0.0	—
	No mutation	19 (86.4)	Others	0.0	0.0	—
LiaF/LiaS/LiaR in *E. faecium* (*n* = 18)	H55Y, A86V, F88L, I119V, V156I, Y224C, I243V/K4R, R7K, L57M, T77A, L100H, L101Q, K117M, V136M, E153K, D251E, S258T, I347T/T45A, E75K, E142D	5 (27.8)	262 (4), 267 (1)	0.0	—	100.0
	H55Y, A86V, F88L, I119V, V156I, Y224C, I243V/K4R, R7K, L57M, T77A, L100H, L101Q, K117M, T120A, V136M, S258T/T45A, W73C, E75K, E142D	4 (22.2)	117 (3), 3096 (1)	25.0	—	75.0
	No mutation	9 (50.0)	17	0.0	—	100.0
MurA in *E. faecalis* (*n* = 22)	V356I	1 (4.5)	41	100.0	0.0	—
	No mutation	21 (95.5)	Others	33.3	42.9	—
MurA in *E. faecium* (*n* = 18)	No mutation	18 (100.0)	All	16.7	16.7	—

^a^ * Amino acid substitutions in PBP5 differing from the predominant group; all other substitutions are identical. SDD, susceptible-dose dependent; I, intermediate; R, resistant; —, no applicable interpretive category.

## Data Availability

This WGS project has been deposited in the Sequence Read Archive (SRA) under BioProject number PRJNA1462655 and BioSample numbers SAMN58963889—SAMN58963928 (Table S4).

## Supplementary Materials

The following supporting information can be downloaded at https://www.mdpi.com/article/10.3390/antibiotics15090867/s1, Figure S1: Distribution of pan-genome gene clusters mapped onto a core gene SNP-based phylogenetic tree of 22 *E. faecalis* isolates and 18 *E. faecium* isolates; Table S1: Metadata, MIC/MBC values, AMR patterns, and biofilm-forming ability of *Enterococcus* spp.; Table S2: The assembly statistics and genome characteristics of the 40 *Enterococcus* spp. isolates; Table S3: The accession numbers of the reference sequences used for the sequence alignment and mutation analysis; Table S4: Accession numbers of WGS data among 40 *Enterococcus* isolates.

## Abbreviations

The following abbreviations are used in this manuscript:

AMR	Antimicrobial resistance
AMP	Ampicillin
ARGs	Antimicrobial resistance genes
BHI	Brain heart infusion
BLAST	Basic Local Alignment Search Tool
BV-BRC	Bacterial and Viral Bioinformatics Resource Center
CARD	Comprehensive Antibiotic Resistance Database
CC	Clonal complex
CDSs	Coding sequences
cgSNPs	Core-genome single-nucleotide polymorphisms
CLSI	Clinical and Laboratory Standards Institute
CPS	Capsular polysaccharide
DAP	Daptomycin
Efm	*Enterococcus faecium*
Efs	*Enterococcus faecalis*
EUCAST	European Committee on Antimicrobial Susceptibility Testing
FOS	Fosfomycin
GEN	Gentamicin
HLGR	High-level gentamicin resistance
I	Intermediate
LZD	Linezolid
MALDI-TOF MS	Matrix-assisted laser desorption/ionization time-of-flight mass spectrometry
MBC	Minimum bactericidal concentration
MDR	Multidrug-resistant
MIC	Minimum inhibitory concentration
MLSB	Macrolides, lincosamides, and streptogramin B
MLST	Multilocus sequence typing
NCBI	National Center for Biotechnology Information
OD	Optical density
ODc	Optical-density cut-off
ORI	Oritavancin
PBS	Phosphate-buffered saline
PBP	Penicillin-binding protein
R	Resistant
S	Susceptible
SDD	Susceptible-dose dependent
SNP	Single-nucleotide polymorphism
ST	Sequence type
TSB	Tryptic soy broth
VAN	Vancomycin
VF	Virulence factor
VFDB	Virulence Factor Database
VFGs	Virulence factor genes
VRE	Vancomycin-resistant enterococci
VREfm	Vancomycin-resistant *Enterococcus faecium*
VREfs	Vancomycin-resistant *Enterococcus faecalis*
WGS	Whole-genome sequencing
